# Supplementary material for: Merging transformation optics with electron-driven photon sources
Source: Nat Commun. 2019 Feb 5;10:599. doi: 10.1038/s41467-019-08488-4 (PMC6363763; doi:10.1038/s41467-019-08488-4)
Supplement: Supplementary file 1 — Supplementary Information [file 41467_2019_8488_MOESM1_ESM.pdf]

Supplementary Information

Merging Transformation Optics with Electron-Driven Photon Sources

Talebi et al.

Supplementary Figures

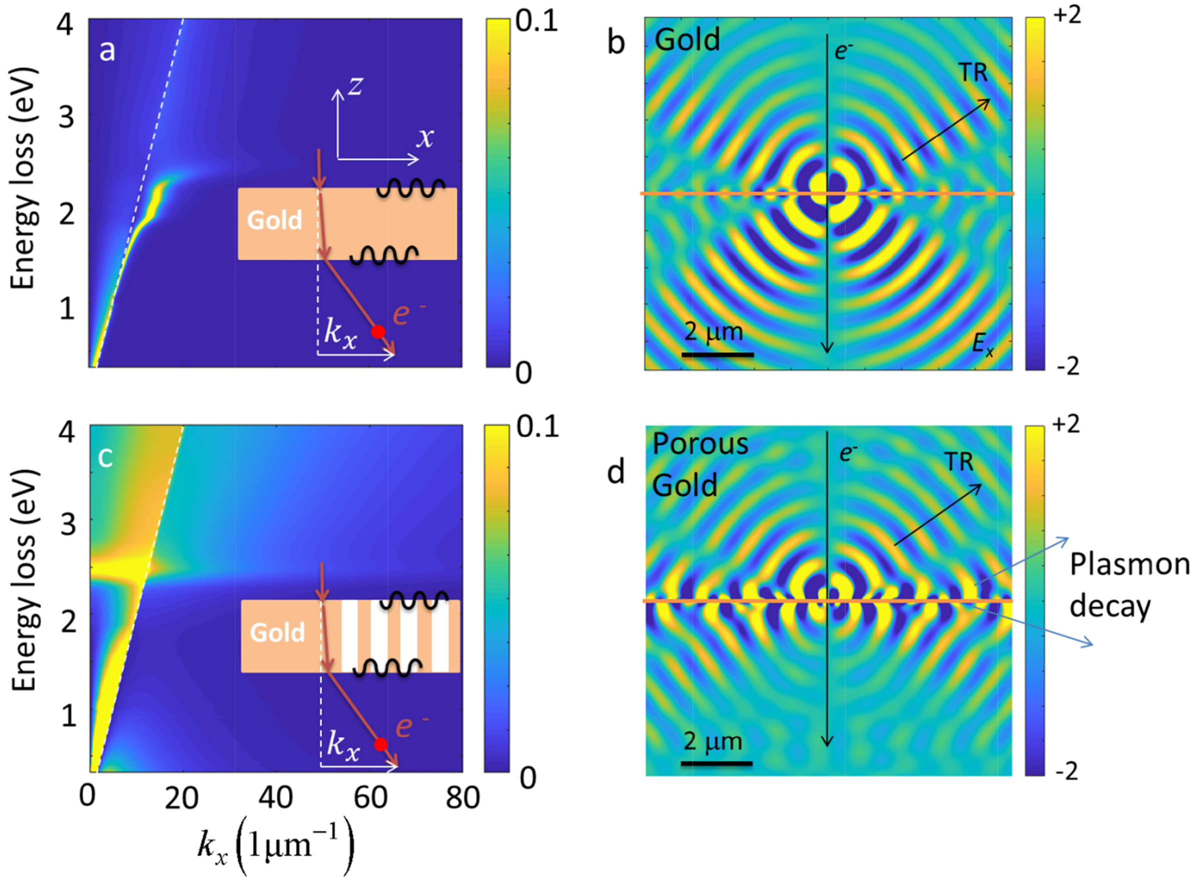

Supplementary Figure 1 Comparing electron-induced SPPs in pure and porous gold thin films. Momentum-resolved EELS and electron-induced radiation (including transition radiation and radiation damping of launched SPPs) for interaction of 30 keV electrons with a uniform 50 nm – thick **a, b** gold film and **c, d** gold film with an inhibited hexagonal lattice of holes. Shown in the **b** and **d** panels are the spatial profiles in the xz plane of the *x*-component of the scattered electric field at a photon energy of $E = 1.55\text{eV}$. Color bar is the EELS intensity at arbitrary units. **a** and **c** panels show the momentum-resolved EELS map.

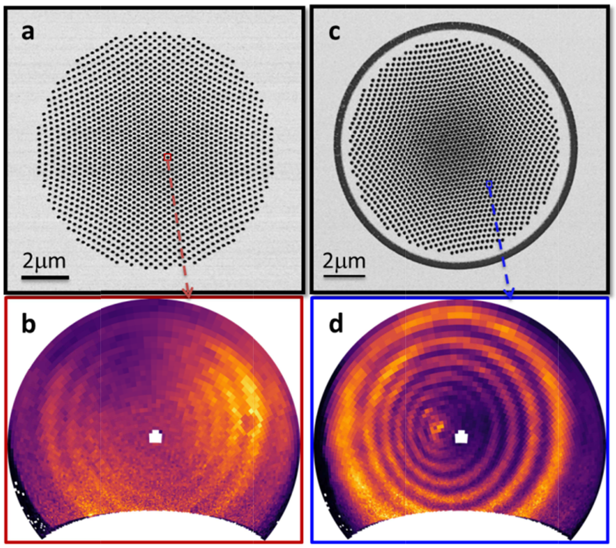

Supplementary Figure 2 Effect of void ring on the generated light field. Dark-field STEM image of the lens and the CL map for structures **a** and **b** without and **c** and **d** with the void ring, respectively (R= 10 μm , *E* = 1.9 eV).

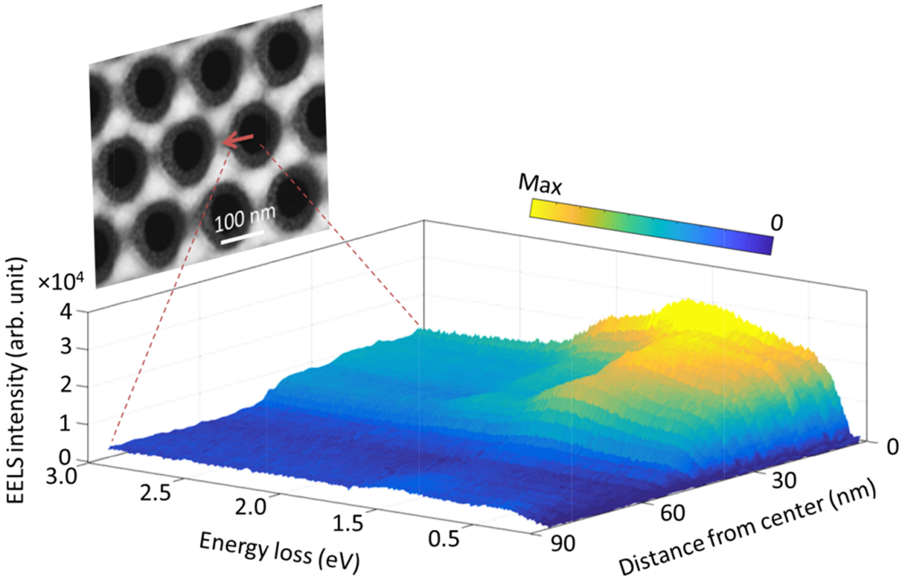

Supplementary Figure 3 EELS spectra along the radius of the central hole. Color scale is the EELS intensity in arbitrary units.

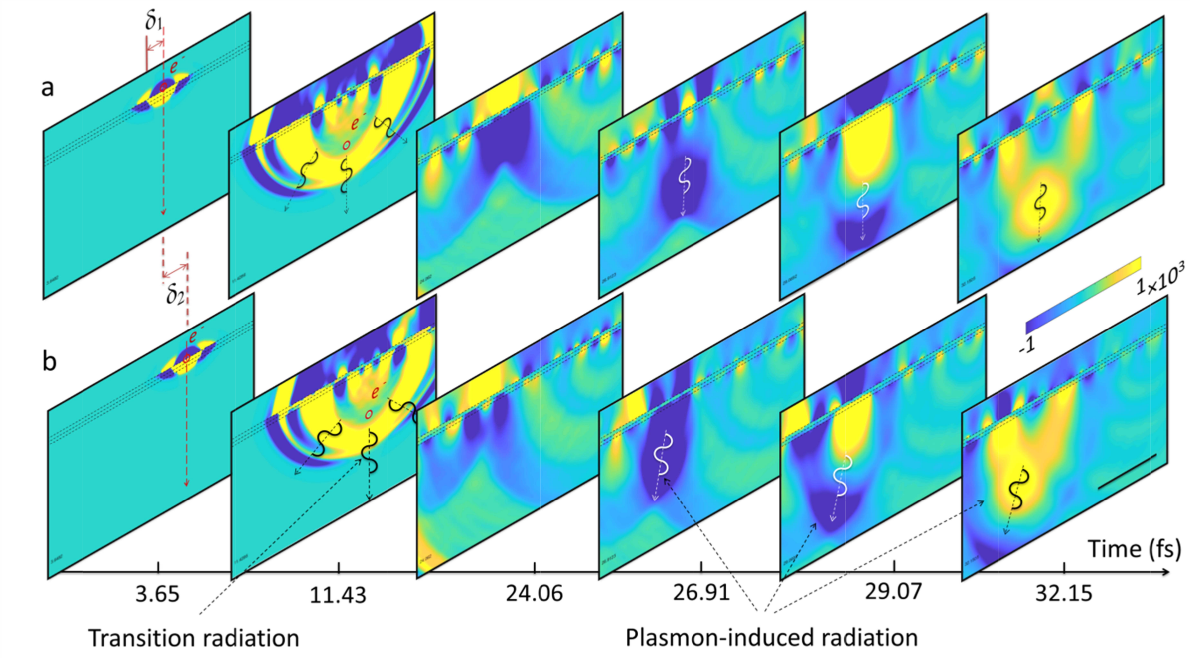

Supplementary Figure 4 Dependence of the angle of the radiation on the distance of the electron beam trajectory from the center of the lens. Snapshots of the *z*-component of the electric field at depicted times for the electron impact position at **a** 400 nm and **b** 1000 nm away from the center ($\delta_1 = 400\,\mathrm{nm}, \delta_2 = 600\,\mathrm{nm}$). The simulations here have been performed for a lens with only 5 µm radius, therefore the dependence of the radiation angle upon the distance from the center is further enhanced in comparison with lens structures proposed in the main manuscript. Color bar is the electric field amplitude in the dimensions of $\mathrm{V\,m^{-1}}$. Scale bar is $2\,\mu\mathrm{m}$.

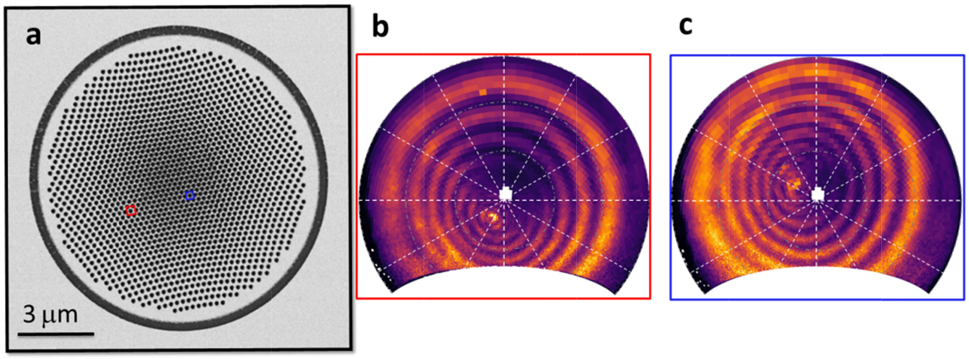

Supplementary Figure 5 Controlling the directionality of the radiation by electron impact. **a** A fabricated planar lens with a focal length of 10 μm. **b** and **c** CL map at E = 1.6 eV at depicted positions (red spot corresponds to CL image **b**, and blue spot corresponds to CL image **c**).

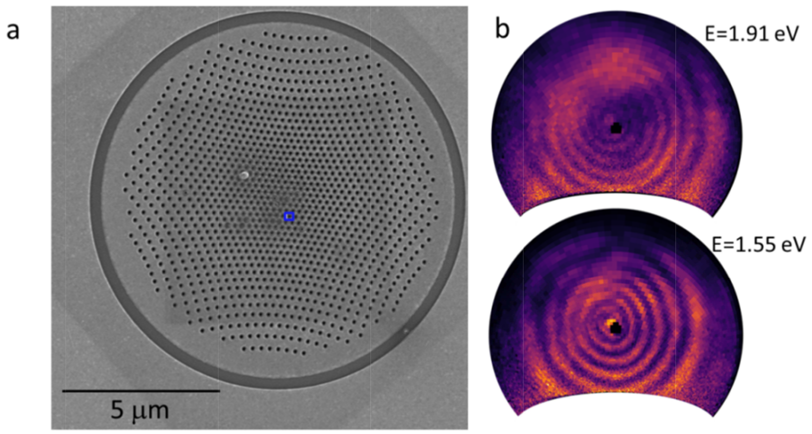

Supplementary Figure 6 Measured CL map at two different photon energies. **a** SEM image of a fabricated planar lens with a focal length of 2 μm. **b** CL map at two different wavelengths of 1.55 eV and 1.91 eV, with the electron beam exciting the structure at the blue spot. The gray spot is due to contamination happening during the measurement.

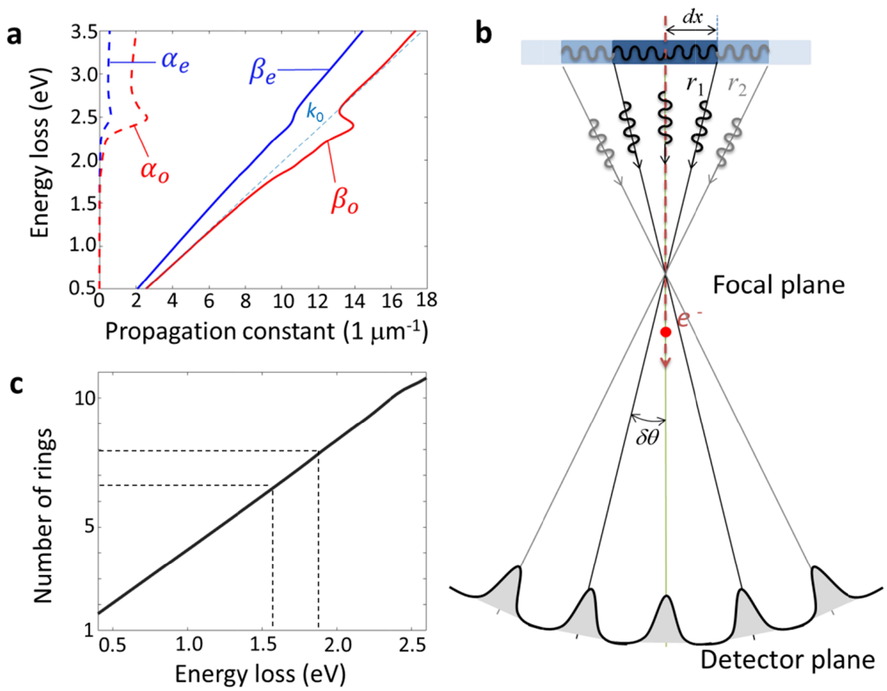

Supplementary Figure 7 Number of far-field rings versus the photon energy. **a** SPP dispersion in a thin film made of gold and a hexagonal lattice of air holes, modelled using an effective medium approach. β is the phase constant and α is the attenuation constant (see Supplementary Section 1). **b** A geometrical consideration for describing the number of intensity maxima observed in the measured CL maps. **c** Calculated number of rings versus photon energy for a lens radius $D = 5.6\,\mu\text{m}$.

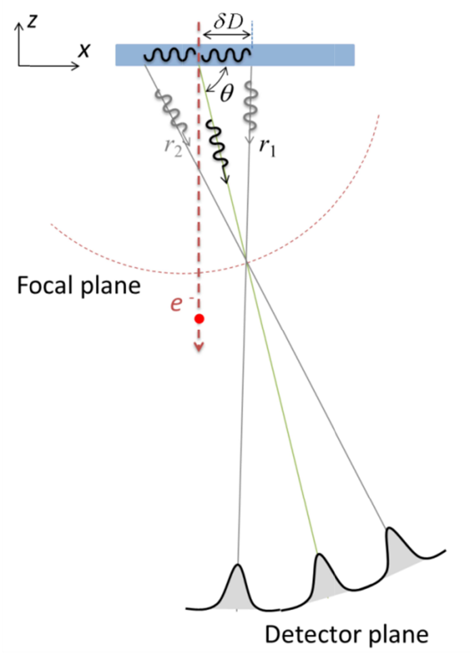

Supplementary Figure 8 Tuning directionality with electron impact. Dependence of the inclination angle of radiation on the asymmetry of the structure with respect to the electron trajectory.

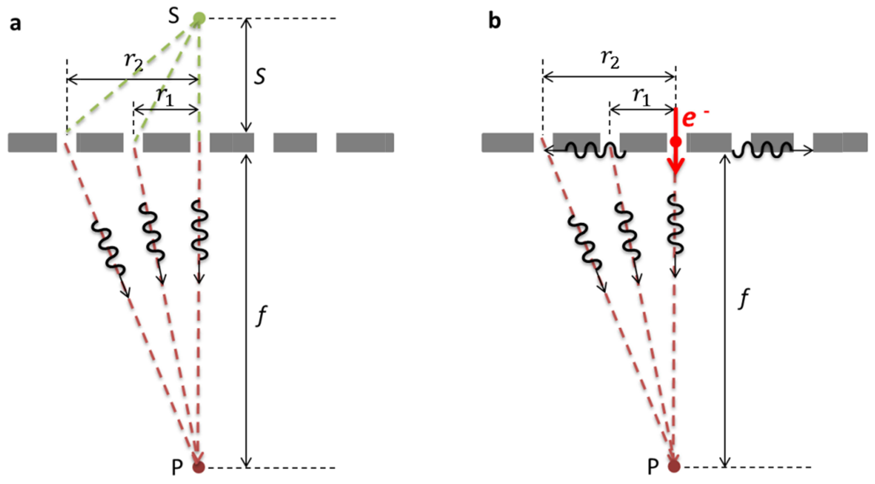

Supplementary Figure 9 Comparing the functionality of photon sieves for far-fied light and electron-induced polaritons. Schematic of the focusing capability of a photon sieve for **a** x-rays, and **b** electron-induced plasmon polaritons.

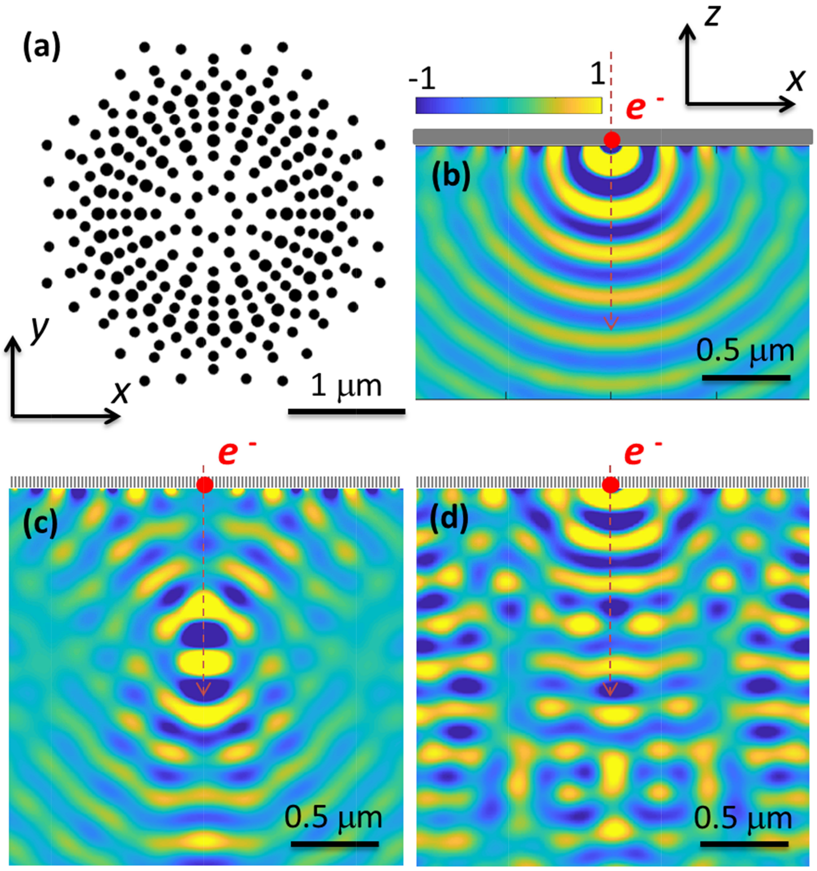

Supplementary Figure 10 Photon Sieve for electron-induced plasmon polaritons. **a** Distribution of holes to achieve focused radiation at $f = 900\,\text{nm}$ and $E = 6\,\text{eV}$, for an Al thin film. The z-component of the electric field at a given time for **b** a non-structured and **c** structured Al film at $E = 6\,\text{eV}$, and **d** for a structured Al film at $E = 6.3\,\text{eV}$. Color bar is the electric field amplitude at arbitrary units.

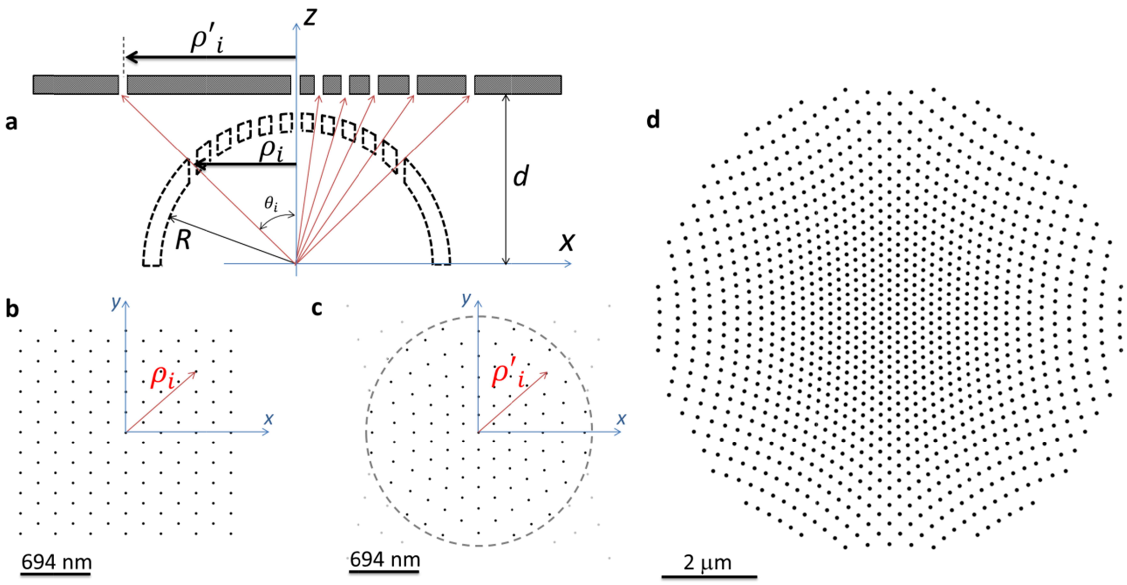

Supplementary Figure 11 Projection algorithm for the designed EDPHS. **a** Mapping principle for projecting the lattice of pinholes from a spherical domain to a planar thin film. The 2-dimensional distribution of the lattice points at the **b** original coordination and **b** final coordination. **c** The final distribution of lattice points for the fabricated EDPHS.

Supplementary note 1 - SPP dispersion and radiation damping in porous thin films

An electron interacting with a thin metal film creates SPPs and loses momentum and energy accordingly. We have calculated the momentum-resolved EELS spectra (MR-EELS) using a previously described method[1]. MR_EELS from a 50-nm thick gold film and 30 keV electrons demonstrates clearly the dispersion of SPPs, as well as a bright and broad peak inside the light cone (Supplementary Figure 1a). The latter is due to transition radiation, which is the dominant mechanism of radiation for electrons interacting with a thin gold film (Supplementary Figure 1c).

As the dimensions of the holes inside the spherical and planar lenses are much smaller than the wavelength of SPPs, an effective medium theory can be used to understand the qualitative behavior of SPPs in such structures. The effective permittivity becomes anisotropic, with $\varepsilon_{\|} = \varepsilon_{xx} = \varepsilon_{yy} = (d_1 + d_2)\varepsilon_{\mathrm{Au}}\varepsilon_{\mathrm{d}} / (d_1\varepsilon_{\mathrm{Au}} + d_2\varepsilon_{\mathrm{d}})$ and $\varepsilon_{\perp} = (d_1\varepsilon_{\mathrm{d}} + d_2\varepsilon_{\mathrm{Au}}) / (d_1 + d_2)$, where d_1 and d_2 are the diameters of the holes and rim-to-rim distances between the holes, respectively[2]. Here, we assumed that the permittivity components parallel to the surface of the thin film are the same; hence the bulk permittivity is modelled with that of a uniaxial crystal. Moreover, the frequency-dependent propagation constant of the ordinary and extraordinary SPP waves propagating at the surface of such materials in general are given as $\gamma_{\mathrm{o}} = \beta_{\mathrm{o}} + i\alpha_{\mathrm{o}} = k_0\sqrt{\varepsilon_0(\varepsilon_0 + 1)^{-1}}$ and $\gamma_{\mathrm{e}} = \beta_{\mathrm{e}} + i\alpha_{\mathrm{e}} = k_0\sqrt{(\varepsilon_{\|}\varepsilon_{\perp} - \varepsilon_{\|})(\varepsilon_{\|}\varepsilon_{\perp} - 1)^{-1}}$, respectively, where β is the phase constant and α is the attenuation constant [3]. For a gold thin film with an inhibited hexagonal lattice of holes, we assume $d_1 = 100\,\mathrm{nm}$ and $d_2 = 50\,\mathrm{nm}$. An electron interacting with this film excites the extraordinary SPP waves, with a bright intensity of the EELS signal inside the light cone (Supplementary Figure 1b). Moreover, the intensity of TR radiation is less than that of the pure gold film, whereas the SPP radiation damping is greatly enhanced (Supplementary Figure 1d). Obviously, the inclination angle of the radiation for a uniform gold film without any discontinuity is towards a direction that is out of reach of the focus point. However, hyperbolic SPPs reaching the void ring will be reflected and hence radiate into the inclination angle required for focusing.

Supplementary note 2 - Effect of the void ring on the response of the lens

In order to enhance the photon generation probability and the contribution of the excited plasmon polaritons to the radiation continuum, a void ring is incorporated around the circumference of the inhibited holes (compare Supplementary Figure 2a to 2c). The inserted ring facilitates the reflection of the SPPs and hence the reflected SPPs will again interact with the structure and contribute to the radiation. The visibility of the maximum intensities in the CL map, demonstrates the interferences which happen due to the reflection of the SPPs from the inserted ring (compare Supplementary Figure 2b to 2d). Note that also for the structure without the void ring the discontinuity in the structure introduced by the termination of the inserted holes by itself enforces reflection of the SPPs, though the effect is smaller.

Supplementary note 3 - Excitation of void plasmons

When the electron beam interacts with a gold thin film at the thickness of 50 nm, the most intense energy loss is expected to occur at an energy loss of 2.6 eV due to the excitation of bulk plasmons. However, we interestingly observe here that localized void plasmons[4] which are supported by the nanohole cavities cause more intense energy-loss signals than bulk plasmons (Supplementary Figure 3). This phenomenon corresponds to the enhanced radiation for the electron beam passing through the holes in comparison with the radiation from bulk plasmons[5] due to the enhanced Purcell factor. Note that the enhanced energy loss is only observed in the holes for a distance near the edge within the near field of the electron beam, as was also observed for CL on holes[6].

Supplementary note 4. Directional radiation from the electron driven photon source

When the electron traverses the structure exactly at the center, we observe the generation of ultrashort electromagnetic pulses which leave the structure and propagate along the direction normal to the surface. Interestingly, when the electron beam excites the structure at a certain distance with respect to the center, an inclined and directional radiation is formed of which the angle is precisely tuned by moving the electron beam further away from the center of the structure (Supplementary Figure 4). This observation is also further confirmed by the experimental angular CL distribution (Supplementary Figure 5).

Supplementary note 5 - Angle resolved CL map at two different energies

The observation of 8 centrosymmetric rings in the angle-resolved CL distribution is observed even for an electron driven photon source with similar geometrical size but different focal length (Supplementary Figure 6a and 6b). This is due to the fact the number of rings is related to the effective size of the structure with respect to the wavelength. The fact that the angle-resolved CL distribution acquired at a higher energy for the same structure and electron impact position exhibits more rings further confirms the dependence of the number of rings on the size of the structure relative to the wavelength.

Supplementary note 6 - Number of rings in the CL map

The radiation from the designed electron-driven photon source is first focused at the focal plane and then diverges, causing an interference pattern in the far-field. The number of the maxima and minima at the detector plane (CL detector) is calculated here. We first notice that the plasmons are gradually radiated from the structure, caused by the engineering of the refractive index. An effective medium theory is used to derive the effective (anisotropic) permittivity of the gold film as discussed in Supplementary section 1. The size of the unit cell is gradually altered versus the distance from the center of the structure (r) (Supplementary Figure 1b). In this way, the location of the nth projected hole from the center of the lens is $R_n = nf\,S\left(f^2 - (nS)^2\right)^{-\frac{1}{2}}$, where $S = 150\,\mathrm{nm}$ is

the lattice size of the initial hexagonal lattice and f is the focal length. We calculate $d_1 = 100\,nm$ and $d_{2n} = R_n - R_{n-1} - d$. The plasmon dispersion for $d_2 = d_1 = 100\,\text{nm}$ for both ordinary and extraordinary waves for a thin film made of an anisotropic material with permittivity values described in supplementary section is calculated (Supplementary Figure 7a). It is apparent that the SPP dispersion is significantly altered by the presence of the holes, and that the plasmon dispersions is shifted into the light cone. Comparing Supplementary Figure 7b and Supplementary Figure 1 shows that the swift electrons are coupling to the extraordinary plasmon waves, and hence the radiation damping is significantly enhanced. Extraordinary plasmon waves are plasmon polaritons propagating in a uniaxial anisotropic material at the direction normal to the optics axis [7]. In other words, the relevant effective mode index is that of the extraordinary plasmons with

$$n_{\text{eff}} = \sqrt{\left(\varepsilon_{\|}\varepsilon_{\perp} - \varepsilon_{\|}\right)\left(\varepsilon_{\|}\varepsilon_{\perp} - 1\right)^{-1}} \quad (1)$$

where n_{eff} is the mode index. We further derive a simple geometrical optics formulation which describes the focusing ability of the lens as well as the number of rings m in the CL map (Supplementary Figure 7b). In order to obtain constructive interference at the focal plane, we should have

$$n_{\text{eff}} k_0\, r + k_0\sqrt{f^2 + r^2} = k_0 f \pm 2m\pi\,, \quad (2)$$

where f is the focal length and r the distance of the propagation which is measured from the center of the structure. This simple phase relation can further be integrated over r to derive m as

$$m(\lambda) = \left| \frac{1}{\lambda D} \int_{r=0}^{D} \left(r\, n_{\text{eff}}(r,\lambda) + \sqrt{f^2 + r^2} - f \right) dr \right|, \quad (3)$$

where D is radius of the lens (5.6 μm). Note that n_{eff} is itself depending on the wavelength λ and distance. The number of rings is approximately linearly dependent on the photon energy (inversely related to the wavelength) (Supplementary Figure 7c) and in perfect agreement with the experimental data, showing m = 7.94 at $E = 1.9\,\text{eV}$ ($\lambda = 652.5\,\text{nm}$) and m = 6.63 at $E = 1.55\,\text{eV}$ ($\lambda = 729.3\,\text{nm}$) (Supplementary Figure 5). Here we used the following parameters: $D = 5.6\,\mu\text{m}$, $f = 2\,\mu\text{m}$, and R = 50 nm. The linear approximation is understood by the dominance of the third term inside the integrand in eq. 3, whereas the first and second terms add up corrections to the phase of the radiation in such a way to facilitate focusing. When the electron beam traverses the lens at a certain distance with respect to the symmetry axis of the lens, the mode index of the SPPs propagating towards $+x$ and $-x$ directions will be different (see Supplementary Figure 7). The criterion for constructive interference will be given by

$$k_0 f + 2n\pi = n_{\text{eff}}^{+}(r,\lambda) k_0 r + k_0 \sqrt{f^2 + r^2 - 2 f r \cos\theta}$$
$$= n_{\text{eff}}^{-}(r,\lambda) k_0 r + k_0 \sqrt{f^2 + r^2 + 2 f r \cos\theta} \quad (4)$$

where θ is the inclination angle of the radiation with respect to the lens axis. By assuming $r \ll f$ eq. (3) is recast as

$$\cos(\theta) = \frac{1}{2}\Delta n_{\text{eff}}\,, \quad (5)$$

where $\Delta n_{\text{eff}} = n_{\text{eff}}^{+} - n_{\text{eff}}^{-}$ is the difference in the effective refractive index of plasmons propagating in the $\pm x$ directions.

Supplementary note 7 - Transformation Optics and Differences with photon sieves

We first discuss the possibilities for designing an EDPHS which operates by the diffraction of propagating plasmons from incorporated holes. There are a few similarities between the structure discussed here and photon sieves. The latter are based on the diffraction of free-space waves by embedded holes in dielectric thin films, and have been established as an efficient way for focusing x-rays (Supplementary Figure 8a)[8]. In the notation of Supplementary Figure 8a, radiation from a point source S is focused at point P, by means of a structured thin film consisting of a distribution of holes. The size of these transmissive pinholes and their distribution are both chosen in such a way to allow for constructive interference of the diffracted rays at the focal point P. To allow for this, the distribution of the pinholes should satisfy $\sqrt{r_n^2 + S^2} + \sqrt{r_n^2 + f^2} = S + f + n\lambda$. Here r_n is the distance of the nth hole from the origin, λ is the wavelength of the source, and n is an integer. Obviously, the focusing ability strongly depends on the wavelength of the source which hinders photon sieves from offering a broadband response.

In a similar sieve geometry for a moving electron interacting with a metallic thin film, we consider excitation of plasmon polaritons at the impact position of the electron (Supplementary Figure 8b). A distribution of pinholes (or other diffractive centers like metamaterial elements or ribs) is embedded to allow for outcoupling of the propagating plasmons, creating a radiation continuum. For constructive interference of the outcoupled beams at the focal plane, the distribution of the diffractive elements (here pinholes) should satisfy

$$n_{\text{eff}} r_n + \sqrt{r_n^2 + f^2} = f + n\lambda \quad (6)$$

where n_{eff} is the effective mode index of the plasmons in the structured thin film. It should be noted that for both cases (photon sieves for either x rays or plasmons), the size of the pinholes should be large enough to allow for either transmissive response or an efficient outcoupling of

the plasmon polaritons; i.e., the diameter of the holes should be comparable to the wavelength of the source or excited plasmons.

In the following we show how the photon sieve design principle can be used to focus the electron-induced radiation. We consider here a 80 nm-thick Al film and use eq. (6) to calculate the required distribution of the pinholes to facilitate focusing at $\lambda = 206.6\,\text{nm}$ ($E = 6\,\text{eV}$). At this wavelength, the 80-nm Al film sustains plasmon polaritons with a mode index of $n_{\text{eff}} = 1.106 + \text{i}0.015$ (for this thickness, plasmon polaritons at the upper and lower surfaces are uncoupled and the even and odd modes are degenerate). Furthermore, the diameter of the pinholes is varied between $D = 60\,\text{nm}$ and $D = 120\,\text{nm}$ (Supplementary Figure 9a). The radiation from a non-structured Al thin film interacting with a moving electron covers a broad angular range (Supplementary Figure 9b), though by imbedding the pinholes with the desired distribution, we achieve focusing at $f = 900\,\text{nm}$ away from the structured film (Supplementary Figure 9c). However, at a slightly different energy of $E = 6.3\,\text{eV}$, the radiation becomes completely unfocused (Supplementary Figure 9d).

Given the unwanted sensitivity of the photon sieves to the wavelength, we exploit other possibilities which avoid such sensitivity from the design principle. As discussed in the main text, focusing capabilities based on geometrical refraction, like focusing by a hemispherical thin film, are inherently broadband in nature. We therefore here exploit principles of transformation optics to generate a distribution of non-transmissive pinholes, with the aim to engineer the effective mode index of plasmons polaritons to mimic the response of a hemisphere. The general assumption is based on adiabatically tuning the periodicity of the lattice of holes, to achieve an engineered mode index as a function of distance from the origin. Using the Jacobian of the transformation matrix Λ for mapping a function from a spherical coordinate system with coordinates (r, θ, φ) to a cylindrical system with coordinates (ρ, φ, z) (with φ and θ the zenithal and azimuthal angles, respectively), the permittivity of the structure in the cylindrical system $\varepsilon' = |\Lambda|^{-1} \Lambda \hat{\varepsilon} \Lambda^T$ is derived from the permittivity of the material in spherical coordinates ($\hat{\varepsilon} = \varepsilon_{\text{Au}} \text{I}$, where ε_{Au} is the permittivity of gold and I is the identity matrix). Specifically, we take $\varepsilon'_{\rho\rho} = \varepsilon_{\text{Au}} \rho^2 / (\rho^2 + d^2)$, $\varepsilon'_{\rho\varphi} = \varepsilon'_{zz} = 0$, and $\varepsilon'_{\varphi\varphi} = \varepsilon_{\text{Au}}$. Formally, such a structure seems practically out of reach, considering that $\varepsilon'_{zz} = 0$. Nevertheless $\varepsilon'_{\rho\rho}$ dominates the propagation of polaritons and can be suitably engineered. For a hemisphere with the focal point at the center, we set for our mapping purpose $d = R$, so that $\varepsilon'_{\rho\rho} = \varepsilon_{\text{Au}} (\rho/R)^2 \left((\rho/R)^2 + 1\right)^{-1} = \varepsilon_{\text{Au}} \sin^2\theta$. At $\rho \gg f$, $\varepsilon'_{\rho\rho} \simeq \varepsilon_{\text{Au}}$. In other words the distance between the holes should be adiabatically increased for increasing distance from the origin, to obtain the pure gold permittivity at $\rho \gg f$. A geometric projection algorithm which maps the position of the holes on the gold hemispherical film onto a thin film located at $z = d = R$ is used to obtain such a distribution of pinholes (see

Supplementary Figure 10a). We assume a hexagonal lattice of holes with centers located at (x_i, y_i) as shown in Supplementary Figure 10b and use the mapping algorithm to obtain the new distribution for the holes in the thin film as $\rho_i' = R\tan\theta_i$ and $\tan\theta_i = \rho_i \Big/ \sqrt{R^2 - \rho_i^2}$ (Supplementary Figure 10c). To maintain an azimuthally symmetric radiation pattern only the projected holes within a certain radius are included in the design.

For $R \to \infty$ the original lattice will be unambiguously maintained in the projected domain as well (a hemisphere film with $R \to \infty$ corresponds to a flat film). Finally, for the EDPHS investigated here, we assumed $R = 5.7\,\mu\text{m}$, for which the lattice in Supplementary Figure 10d is obtained. At each lattice point a pinhole with a diameter of 100 nm is considered. The overall lattice is incorporated onto an Au thin film with a thickness of 50 nm.

Supplementary References

[1] Talebi N. Interaction of electron beams with optical nanostructures and metamaterials: from coherent photon sources towards shaping the wave function, *J. Opt.* **19**, 103001 (2017).
[2] Kidwai O., Zhukovsky S.V. and Sipe J.E. Effective-medium approach to planar multilayer hyperbolic metamaterials: Strengths and limitations, *Phys. Rev. A* **85**, 053842 (2012).
[3] Liscidini M. & Sipe J. E. Quasiguided surface plasmon excitations in anisotropic materials, *Phys. Rev. B* **81**, 115335 (2010).
[4] Cole R. M., Baumberg J. J., Garcia de Abajo F. J., Mahajan S., Abdelsalam M. & Bartlett P. N. Understanding Plasmons in Nanoscale Voids, Nano Lett. **7**, 2094 (2007).
[5] Arakawa E. T., Birkhoff R. D. & Herickhoff R. J. Detection of Plasma Radiation from Electron-Bombarded Al and Mg Foils, *Phys. Rev. Lett.* **12**, 319 (1964).
[6] Coenen T. & Polman A. Optical Properties of Single Plasmonic Holes Probed with Local Electron Beam Excitation, *ACS Nano* **8**, 7350 (2014).
[7] Talebi N. Optical modes in slab waveguides with magnetoelectric effect *J. Opt.* **18**, 055607 (2016).
[8] Kipp, L. et al. Sharper images by focusing soft X-rays with photon sieves. *Nature* **414**, 184-188 (2001).
