# Supplementary material for: Merging transformation optics with electron-driven photon sources
Source: Nat Commun. 2019 Feb 5;10:599. doi: 10.1038/s41467-019-08488-4 (PMC6363763; doi:10.1038/s41467-019-08488-4)
Supplement: Supplementary file 3 — Description of Additional Supplementary Files [file 41467_2019_8488_MOESM3_ESM.docx]

**Description of Additional Supplementary Files**

File Name: Supplementary Movie 1

Description: Electrons interacting with the designed EDPHS generate an ultra-fast directional light pulse, which is focused at the distance of 5.7 micrometer from the EDPHS. Demonstrated is the dynamics of the z-component of the electric field
